# Progress towards the Development of a Universal Influenza Vaccine

**DOI:** 10.3390/v14081684

**Published:** 2022-07-30

**Authors:** Wen-Chien Wang, Ekramy E. Sayedahmed, Suryaprakash Sambhara, Suresh K. Mittal

**Affiliations:** 1 Department of Comparative Pathobiology, Purdue Institute for Immunology, Inflammation and Infectious Disease, and Purdue University Center for Cancer Research, College of Veterinary Medicine, Purdue University, West Lafayette, IN 47907, USA; wang5382@purdue.edu (W.-C.W.); esayedah@purdue.edu (E.E.S.); 2 Influenza Division, National Center for Immunization and Respiratory Diseases, Centers for Disease Control and Prevention, Atlanta, GA 30329, USA

**Keywords:** universal influenza vaccine, broadly protective influenza vaccine, pandemic preparedness, pandemic influenza vaccine, influenza vaccine, conserved influenza antigens

## Abstract

Influenza viruses are responsible for millions of cases globally and significantly threaten public health. Since pandemic and zoonotic influenza viruses have emerged in the last 20 years and some of the viruses have resulted in high mortality in humans, a universal influenza vaccine is needed to provide comprehensive protection against a wide range of influenza viruses. Current seasonal influenza vaccines provide strain-specific protection and are less effective against mismatched strains. The rapid antigenic drift and shift in influenza viruses resulted in time-consuming surveillance and uncertainty in the vaccine protection efficacy. Most recent universal influenza vaccine studies target the conserved antigen domains of the viral surface glycoproteins and internal proteins to provide broader protection. Following the development of advanced vaccine technologies, several innovative strategies and vaccine platforms are being explored to generate robust cross-protective immunity. This review provides the latest progress in the development of universal influenza vaccines.

## 1. Introduction

Influenza is a highly transmissible viral infection resulting in severe respiratory illnesses in approximately 3 to 5 million people and 290,000 to 650,000 deaths globally every year [1]. Seasonal influenza infections occur in all age groups; however, young children under 59 months, adults over 65 years, and pregnant women are more vulnerable [2,3,4]. Therefore, influenza infections significantly burden the healthcare system in several countries [5,6]. Although considerable efforts have been made to prevent influenza infections, several pandemics have occurred over the past hundred years. These include 1918 A(H1N1) “Spanish flu”, 1957 A(H2N2) “Asian flu”, 1968 A(H3N2) “Hong Kong flu”, 1977 A(H1N1) “Russian flu”, and 2009 A(H1N1)pdm09 “pandemic flu” [7,8]. In addition, emerging zoonotic avian influenza viruses, including A(H3N2), A(H5N1), A(H5N6), A(H7N1), A(H7N3), A(H7N7), A(H7N9), and A(H9N2) subtypes pose a potential pandemic risk [9,10,11,12,13,14].

**Figure 1:**
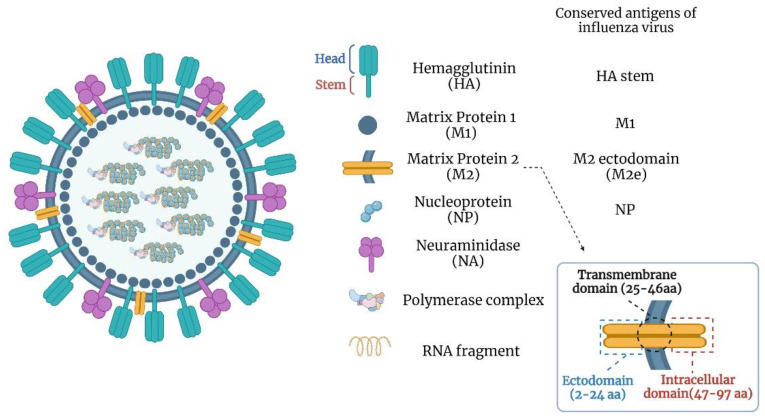
Structural representation of influenza A virus particle and its components.

Influenza viruses have single-stranded, negative-sense segmented RNA genomes and belong to the *Orthomyxoviridae* family. They are classified into four genera, A, B, C, and D, based on the antigenic differences in the nucleoprotein (NP) and matrix 1 (M1) protein. The influenza A genus is highly diverse and has a wide host range. Influenza B infects humans, influenza C infects humans and swine, and influenza D infects cattle and swine. Primarily, influenza A and B viruses pose a significant public health threat. Influenza A and B viruses have 8 RNA segments coding for at least 11 proteins (Figure 1) [15,16,17,18]. The virus envelope consists of hemagglutinin (HA) and neuraminidase (NA) proteins. HA is cleaved into HA1 and HA2, forming the globular-shaped trimers on the viral envelope [19,20]. The virus enters susceptible cells through HA-mediated binding to host receptors following the cell membrane fusion. Since most of the neutralizing antibody targets are in the HA globular head domain, this region is an essential target for conventional influenza vaccines [21,22].

Despite a high incidence of annual seasonal influenza infections, vaccination is still considered an effective strategy to prevent influenza in humans. Currently, the seasonal influenza vaccines are produced as “trivalent” or “quadrivalent” formulations, containing components of A(H1N1)pdm09 and A(H3N2) influenza A viruses and either one or two influenza B viruses (Victoria or Yamagata lineages) [23,24]. For the 2021–2022 influenza season, three types of vaccines were licensed in the United States, including inactivated, recombinant, and live-attenuated vaccines [25]. The inactivated vaccine can be produced in two ways: whole inactivated virus (WIV) [26] and split virus [27]. Since the WIV vaccine often results in adverse effects such as inflammation at the site of injection and fever in children [26,27], split virus inactivation is the primary approach for inactivated influenza vaccines. The virus sources for split vaccines are either embryonated chicken eggs or the Madin Darby Canine Kidney (MDCK) cell line. The split virus vaccine is prepared by disrupting viral particles with a detergent or diethyl ether and purification/enrichment of the HA fraction [25,27]. The recombinant influenza vaccine is based on the baculovirus vector system to express HA on the surface of insect cells [28]. The live attenuated vaccine is designed as cold-adaptive (ca) and temperature-sensitive (ts) for intranasal administration [29]. Due to the high levels of influenza A and B virus replication in eggs, the availability of established infrastructure, and economic considerations, most seasonal influenza vaccines are still mass-produced in embryonated chicken eggs [23].

There are several shortcomings of the current seasonal influenza vaccines. They must be tailor-made yearly to match the expected circulating strains during the influenza season, since influenza viruses undergo antigenic evolution or antigen drift and shift. The selection of influenza virus strains for seasonal influenza vaccines is made for the northern hemisphere in February and the southern hemisphere in September by the World Health Organization (WHO) to allow sufficient time for vaccine production [30]. Nevertheless, the mismatch between the vaccine and circulating strains results in variable protection [31,32,33]. The over-dependence of the current influenza vaccine production system in embryonated hen eggs presents specific challenges, including a relatively longer timeframe from strain selection to vaccine availability, difficulty in scaling up vaccine production in a pandemic situation due to a limited supply of eggs, and the failure of relevant influenza virus strains’ replication to high titers in eggs [34,35,36].

Reports of human infections with either low or highly pathogenic avian influenza (HPAI) viruses of A(H5), A(H7), and A(H9) subtypes underscore the public health threat and pandemic potential posed by these avian influenza viruses (AIV). Since their emergence in Asia over two decades ago, HPAI A(H5N1) viruses have spread to over sixty countries on three continents and are endemic among poultry in Southeast Asia and Africa [37]. Additionally, A(H9N2) infections are enzootic among poultry globally and infect humans sporadically. Both the low and highly pathogenic AIVs of the A(H7) subtype [i.e., A(H7N2), A(H7N3), and A(H7N7)] continue to cause sporadic infections in humans. In 2013, a new A(H7N9) AIV strain of the subtype emerged in China and had caused more than 1568 human infections and 616 deaths as of 5 May 2022 [38]. In 2015, widespread HPAI A(H5N2) virus infections in 15 states of the U.S. resulted in the destruction of approximately 50 million poultry with an estimated USD 3.3 billion in losses [39]. The recent detection of a canine A(H3N2) virus and a feline A(H7N2) virus in the US has further complicated the threat posed by emerging influenza viruses. A genotype 4 Eurasian avian-like reassortant A(H1N1) swine influenza virus with pandemic potential has been identified in China [40].

Although the transmission of avian influenza viruses such as A(H5N1), A(H7N7), A(H7N9), or A(H9N2) viruses have been infrequent and limited in humans, genetic reassortment can occur between any of these AIVs and a circulating human influenza virus. Acquiring crucial mutations in the HA and other genes can confer a binding capability to human-like, α2–6-linked sialic acid receptors [41,42]. These events could result in the generation of a novel pandemic influenza virus with the capacity to infect and effectively transmit among humans with little or no immunity to this new virus.

## 2. The Need for a Universal Influenza Vaccine

The above-described events underscore the significance of developing an immunogenic and effective universal influenza vaccine for influenza pandemic preparedness since the exact features of the pandemic influenza virus will be only known at the start. As a part of pandemic preparedness, vaccines against A(H5N1), A(H7N7), and A(H9N2) viruses have been developed and clinically evaluated; A(H5N1) vaccines have been stockpiled by national health agencies [43,44]. Further, the A(H5N1) virus has been diversified into genetically distinct clades, subclades, and third-order clades, making it extremely difficult to develop subtype-specific vaccines using traditional methods. Similar concerns exist with vaccines developed against A(H7N7), A(H7N9), and A(H9N2) viruses. Furthermore, low cross-reactivity against heterologous viruses, despite the inclusion of MF59 adjuvant, has been reported in clinical trials with A(H9N2) vaccines [45]. Moreover, vaccines against AIVs are often poorly immunogenic. For example, an adjuvant must be used to enhance and broaden neutralizing antibody responses elicited by the A(H5N1) virus inactivated, split, or subunit vaccines [43,44]. The cell culture-derived A(H7N1) vaccine was poorly immunogenic in humans without a suitable adjuvant [46,47,48]. The development of effective antivirals against influenza viruses has been hampered due to their lower efficacy, generation of drug-resistant mutants, and significant side effects in vulnerable populations.

**Figure 2:**
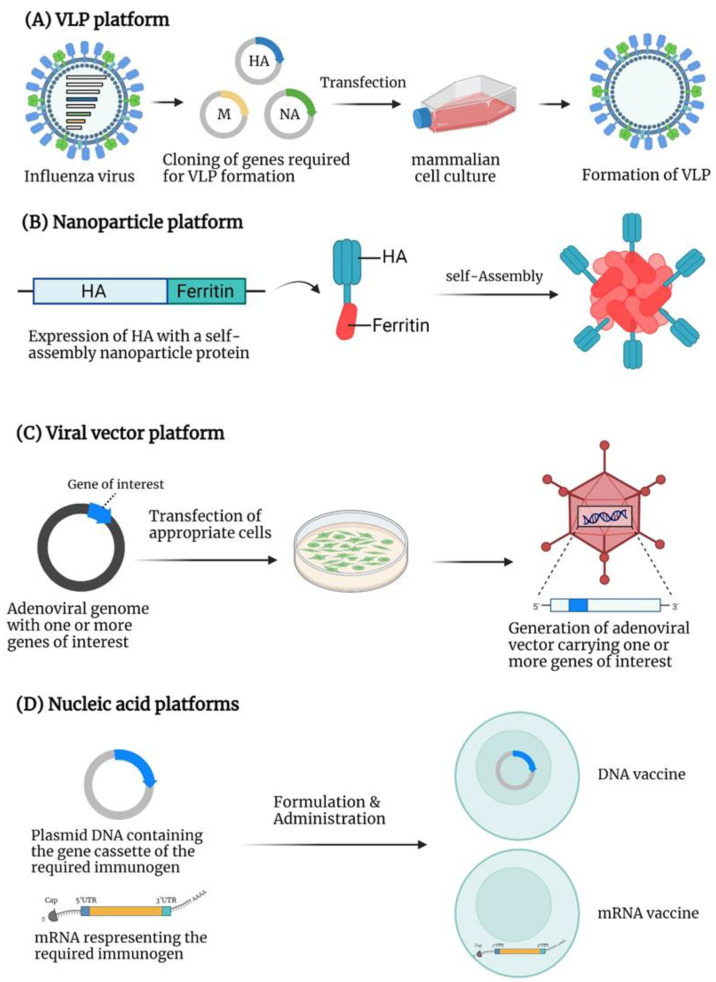
The platforms for universal influenza vaccine development: (**A**) VLP platform: Co-expression of HA, NA, and M gene cassettes of influenza virus generate the VLP of influenza virus. (**B**) Nanoparticle platform: Some proteins or chemical molecules self-assemble into nanoparticles. The figure illustrates that, when the ferritin is expressed with the HA of influenza virus, nanoparticles are formed due to the self-assembly of ferritin displaying multiple HA on the surface. (**C**) Viral vector platform: A plasmid containing the adenoviral genomic sequences with a gene cassette of the required immunogen is constructed. The transfection of an appropriate cell line with the adenoviral genomic plasmid will generate the infectious adenoviral vector expressing the gene of interest. (**D**) Nucleic acid-based platforms: For a DNA vaccine, the plasmid carrying the gene cassette of the required immunogen is constructed and formulated for delivery into a host. Once the plasmid DNA reaches the nucleus, the gene of interest will be expressed. For an mRNA vaccine, the mRNA representing an immunogen is flanked with 5′UTR and 3 UTR and is associated with a 5′ cap and a poly-A tail. The mRNA vaccine is formulated with appropriate materials such as lipid nanoparticles and delivered into the host cells to synthesize the desired immunogen.

The correlates of protection for seasonal influenza vaccines are virus-neutralizing or hemagglutination-inhibition (HI) antibodies against the immunodominant globular head domain of the HA [49]. Due to the presence of hypervariable epitopes in the head domain, the resultant immune response is predominately strain-specific. Hence, newer vaccine platforms are needed with the potential to induce both humoral and cellular immune responses that confer protection against a broad range of influenza viruses emerging from avian or animal reservoirs. Universal influenza vaccine candidates should provide a homosubtypic immunity (immunity against same-subtype viruses, i.e., H1 vaccine protects against H1 viruses) and a heterosubtypic immunity (immunity against different-subtype viruses, i.e., H1 vaccine protects against H5, H7... viruses). This review discusses various vaccine strategies of universal influenza vaccine development, including the conserved antigens of influenza viruses and novel platforms demonstrating the broad immunity against heterologous strains (Figure 2, Table 1).

## 3. Universal Influenza Vaccine Targets

### 3.1. HA Stalk Domain

The entire HA2 and some portions of the N- and C-terminals of HA1 constitute the membrane-proximal stem domain of the influenza virus and play an essential role in cell membrane fusion [50,51]. Lower immune pressure makes the HA stem domain relatively conserved compared to the head domain. Since monoclonal antibodies targeting this domain can provide broad protection in mice [52,53], it is considered a potential target for universal influenza vaccine development. Additionally, the HA can be categorized into two groups based on phylogenetic characteristics. Group 1 contains H1, H2, H5, H6, H8, H9, H11, H12, H13, and H16, and group 2 contains H3, H4, H7, H10, H14, and H15. The cross-protective antibodies toward the stem domain usually cross-react with the members within the same group [54]. Still, the HA-stem antibodies showed relatively lower neutralizing ability than the HA-head-specific antibodies [55].

### 3.2. Matrix Protein 2 Ectodomain (M2e)

The matrix protein 2 (M2) is produced by translating spliced mRNA derived from the influenza gene segment 7 [56]. It is a type III integral membrane protein with 97 amino acids. M2 protein can be activated with acidic pH and forms a proton-selective ion channel [56]. It plays a vital role in viral replication, morphogenesis, and assembly [56]. The M2 protein consists of three domains, including the extracellular N-terminal domain (M2e, residues 2–24), the transmembrane domain (residues 25–46), and the intracellular C-terminal domain (residues 47–97). The first nine amino acids of the M2e are almost identical in influenza A viruses. The high conservation of M2e makes it suitable as a universal vaccine target. There is a weak immune response against M2e during influenza infection or conventional vaccination, perhaps due to steric hindrance by anti-HA antibodies limiting the access of M2e to the immune system [57,58,59,60]. A study indicated that the anti-M2e monoclonal antibody (14C2) can reduce the viral replication of some influenza viruses [61,62]. Although the M2-specific antibodies lack the virus neutralization capability, they can mediate antibody-dependent cell cytotoxicity (ADCC) or complement-dependent cytolysis (CDC) of the virus-infected cells [62,63]. The mild immunity against M2e poses a shortcoming to developing an effective vaccine; therefore, a carrier platform or adjuvant is necessary for improved M2e vaccines.

### 3.3. Nucleoprotein (NP)

The influenza virus nucleoprotein (NP) is encoded by segment 5 of the virus genome and serves as an RNA-binding protein [64]. It is associated with viral RNA (vRNA) and forms the ribonucleoprotein (RNP) complex together with the polymerase complex (PB1, PB2, and PA proteins). The RNP complex is essential for viral replication and transcription [65]. The NP is relatively conserved in influenza A viruses with less than 11% difference in amino acid residues [66]. It is a critical target for the cross-reactive cytotoxic T lymphocytes’ (CTL) response against influenza A viruses [67,68,69]. The CTL response is vital for the recovery from influenza infection by destroying the virus-infected cells [69,70]. Due to sequence conservation and the development of broad NP-specific immunity, NP offers an attractive target for universal influenza vaccine development.

### 3.4. Neuraminidase (NA)

Neuraminidase (NA) is a tetrameric glycoprotein on the viral surface and plays a critical role in viral replication [71]. It functions as a sialic acid cleaving enzyme to release the newly produced influenza viruses [72]. Some antiviral drugs, including oseltamivir, laninamivir, peramivir, and zanamivir, target NA and are effective for both influenza A and B viruses [71,73]. Antibodies against NA can reduce the duration of influenza illness and impact virus transmission [74]. Moreover, anti-NA antibodies are believed to protect with a mechanism that is different from HA [75]. NA undergoes antigenic drift and shift; however, NA has slower antigenic evolution than HA [76]. The NA enzymatic site located between 222 and 230 amino acid residues is highly conserved among influenza A and B viruses [77]. A monoclonal antibody targeting this epitope could inhibit N1–N9 influenza viruses [78], suggesting its significance for a universal influenza vaccine strategy.

## 4. Virus-like Particle (VLP)-Based Vaccines

VLPs are non-infectious macromolecular structures from the self-assembly of viral proteins without viral genetic materials. VLPs present the viral antigens in the unmodified configuration on the surface, similar to viruses [79].

**Figure 3:**
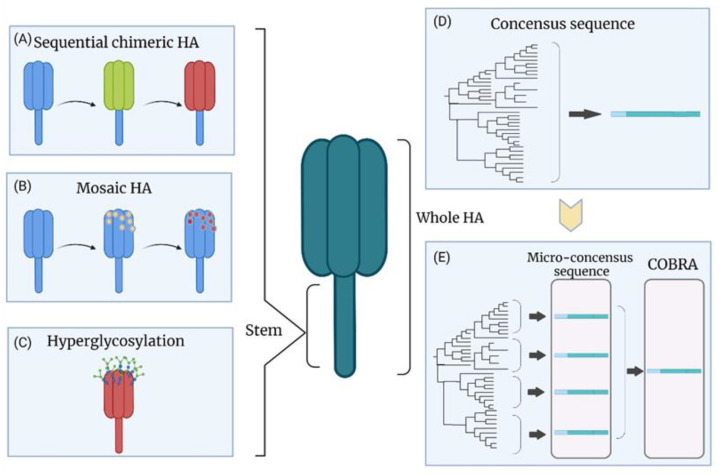
HA-specific approaches for universal influenza vaccines: (**A**) Sequential immunization with chimeric HA head: The strategy is based on the multiple HAs to use an identical stem domain with different exotic head domains. The robust immune response against the HA stem is generated by sequential administration. (**B**) Sequential immunization with mosaic HA head: The major immunogenic sites of the head domain are replaced with the sequences from exotic viral strains leading to an enhanced immune response against the HA stem domain. (**C**) Hyperglycosylation of HA head: The HA hyperglycosylation shields the antigenic sites from recognition by neutralizing antibodies. It may lead to better recognition of the HA stem domain-specific epitopes. (**D**) Consensus HA sequence: The consensus HA sequence is generated by aligning HA protein sequences from the database. The HA consensus protein may provide broad protection. (**E**) Micro-consensus HA sequence and COBRA: Micro-consensus sequences are based on the consensus sequences from each branch of a phylogenetic tree. The cocktail administration can provide better efficacy in highly diverse HA populations. COBRA sequence is generated by multiple rounds of the consensus sequence procedure, thereby reducing the sampling bias.

Computationally optimized broadly cross-reactive antigen (COBRA) (Figure 3D,E) is a strategy that can generate a consensus HA for VLPs. Briefly, COBRA is based on multiple rounds of consensus sequence generation. The shared amino acids are chosen and constructed to be the consensus sequence with the alignment of HA protein sequences from the database or sampling. The multiple-round processing minimizes the sampling bias by excluding the single outliers from the database [80,81,82]. The influenza VLP-based vaccine by COBRA was developed against the A(H5N1) virus in the lentiviral VLP system [83]. The COBRA HA maintained its natural functions similar to the wild-type HA, and the vaccine provided complete protection against the homologous A(H5N1) virus in mice, ferrets, and cynomolgus macaques [83,84]. Mice immunized with the COBRA H1N1 HA VLP vaccine showed only mild morbidity with no mortality after challenge with different A(H1N1) viruses (A/California/07/2009 in BALB/c mice and A/Brisbane/02/2018 in DBA/2J mice), and there was a decrease in viral lung load [82]. The serum samples from immunized mice showed high HI titers against several A(H1N1) viruses [82]. Another study was based on developing several COBRA VLPs using HA sequences from A(H1N1) viruses from the past 100 years. Four VLPs were selected as vaccine candidates due to their broader HI activities. The candidates were delivered in a cocktail combination or a prime-boost immunization [85]. The group primed with a VLP of pandemic H1 strains and boosted with VLP of modern seasonal H1 strains showed wide protective HI titers. The broad HI activities elicited by these VLP candidates were also observed in ferrets [85,86]. Several COBRA VLPs targeting the A(H3) HA sequences from 2002 to 2014 were produced as vaccine candidates [87]. The VLPs consisting of 2002–2005 HA sequences showed broad protection against A(H3N2) viruses from 2002 to 2007, and several COBRA VLPs targeting 2009–2015 HA sequences expressed higher neutralizing antibody titers against A(H3N2) viruses from 2012 to 2016 compared to the wild-type HA VLPs [87]. Overall, COBRA provides a powerful platform to reduce the impact of antigenic drift and is an alternative approach designed to break the obstacle of the conventional vaccines’ narrow protection.

While the COBRA VLP strategy showed subtype-specific cross-protection, it did not reach the level of a potential universal influenza vaccine. However, several VLPs having COBRA HAs for either group 1 (H1, H8, H13) or 2 (H3, H4, H10) were generated, and BALB/c mice were immunized with four different combinations [88]. The groups immunized sequentially with three doses of groups 1 and 2 HA VLPs elicited high levels of humoral and cell-mediated immune responses. Immunized animals were fully protected from homosubtypic and heterosubtypic influenza viruses [88]. A prime-boost immunization of BALB/c mice with a cocktail of HA VLPs (H1+H3+H5+H7) conferred broad protection following challenge with several homosubtypic and heterosubtypic influenza viruses [89]. However, the heterosubtypic challenge six months after immunization showed 20% mortality, indicating that the protection may be of short duration. In addition, the 40% mortality in immunized aged mice after the heterosubtypic challenge implied suboptimal immune responses in the elder mice [89].

Five copies of different M2e from human, swine, and avian influenza viruses (M2e5x) were used in a VLP format [90]. The study demonstrated that VLPs containing the tandem M2e showed better immune responses and long-lasting protection than VLPs with a single copy of M2e [90,91]. The immunized BALB/c mice showed cross-protection against A(H1N1)pdm09 and A(H3N2) influenza viruses. Moreover, the antibody titer and protection remained high after eight months post-immunization [90]. Recently, heterosubtypic 3xM2e VLPs with trimer-HA stalks (AP-3xM2e/tri-stalk) were generated, and immunization with AP-3xM2e/tri-stalk led to complete protection against A(H1N1) and A(H3N2) viruses in BALB/c mice [92].

## 5. Nanoparticle-Based Vaccines

Several molecules such as polymers, ferritin, liposomes, or metal particles are utilized in designing nanoparticle-based vaccines [93,94]. Nanoparticles are an effective vehicle for presenting targeted immunogens to antigen-presenting cells and inducing B and T cell-based immune responses. [93,95]. Small nanoparticles (20–200 nm in diameter) can effectively diffuse into the lymphatic system. The resident macrophages and dendritic cells will execute the antigen-presenting functions to stimulate antigen-specific immune responses. Large nanoparticles (500–2000 nm in diameter) are primarily trapped outside the lymphatic system and only allow the antigen presentation by the dendritic cells at the injection sites [96,97,98].

The intracellular iron storage protein ferritin can self-assemble as nanoparticles [94]. The A(H1N1) HA of A/New Caledonia/20/1999 was fused to the *Helicobacter pylori* ferritin leading to the presentation of eight trimeric HA domains on the surface of ferritin nanoparticles (HA-np) [99]. High levels of virus-neutralizing antibodies against A(H1N1) viruses from 1934 to 2007 were observed in BALB/c mice immunized with two inoculations. The vaccinated ferrets in this study were also fully protected from the challenge of unmatched A(H1N1) viruses [99]. Interestingly, antibodies were elicited against the receptor binding site (RBS), and the conserved HA stem region led to broader protection [99]. Mosaic nanoparticles displaying hypervariable RBS of multiple HAs resulted in a more comprehensive response against H1N1 influenza viruses spanning over 90 years [100].

The HAs linked to a computationally designed nanoparticle component, I53_dn5B, were generated. The I53_dn5B with I53_dn5A assembles in vitro and forms a nanoparticle presenting 20 HA trimers on its surface [101]. Due to the incorporation of a high number of immunogens in a nanoparticle, four subtypes of HAs from H1, H3, and influenza B viruses were co-expressed on the mosaic nanoparticles [101]. The vaccine induced higher levels of humoral immune responses against several homosubtypic and heterosubtypic (H5, H6, H7, H10) viruses compared to the conventional quadrivalent inactivated vaccine and showed greater protection after challenge with a distant A(H5N1) virus [101]. In another study, the trivalent HAs were assembled with the detergent PS80 to form nanoparticles and were used as a vaccine candidate with the saponin-based adjuvant [102]. It induced higher neutralizing antibody titers against homologous viruses than the trivalent inactivated vaccine and provided broad antibody responses to historic A(H3N2) viruses in ferrets [102]. Tandem M2e domains associated with human, swine, avian, and domestic fowl influenza A viruses were assembled as nanoparticles by ethanol desolvation [103]. The outer layer of the nanoparticle was further coated with M2e-NA fusion tetrameric proteins. This bilayer nanoparticle vaccine stimulated protection against different homologous and heterosubtypic viruses in BALB/c mice with prime-boost immunization and showed long-term protection (4 months) [103].

Polymer-based nanoparticles also provide adjuvant-like functions. For instance, chitosan, a natural polymer, is biodegradable, non-toxic, and appropriate for mucosal administration [104], stimulating the mucosal lymphoid tissues and enhancing the uptake of immunogen [105,106,107]. The conserved M2 domain fused with HA2 and cholera toxin subunit A1 were encapsulated into poly-γ-glutamic acid (γ-PGA)-chitosan nanoparticles [108]. Intranasal immunization of BALB/c mice resulted in protection against several heterosubtypic influenza viruses [108]. The inactivated swine influenza vaccine encapsulated in chitosan elicited cross-reactive mucosal and cellular immune responses against homologous and heterosubtypic influenza viruses in pigs [109]. Another polymer, polylactic-co-glycolic acid (PLGA), was used to produce a swine influenza nanoparticle-based vaccine. It reduced the viral load in immunized pigs following the heterologous virus challenge [110].

## 6. Viral Vector-Based Vaccines

A replicative-defective human adenoviral (Ad) type 5 (HAd5) vector expressing the NP of influenza A/PR/8/34(H1N1) and consensus M2 was used as a single-dose intranasal vaccine. The immunized BALB/cAnNCr mice were completely protected from challenges with A(H1N1), A(H3N2), and A(H5N1) influenza viruses, and the protection lasted for ten months [111]. The vaccine conferred cross-protection against HA groups 1 and 2 influenza A viruses without needing a booster [112,113]. Similarly, the HAd5 vector expressing NP of B/Yamagata/16/88 was used as a single-dose intranasal vaccine, leading to a higher CD8+ immune response and better protection than the intramuscular route in BALB/c mice, suggesting that CD8 T cells play a critical role in protection [114]. Another study gathered most of the conserved domains, including the M2 ectodomain (M2e), HA fusion domain, NP T-cell epitope, and HA α-helix domain of an A(H5N1) virus expressed in the HAd5 vector platform [115]. As expected, this vaccine did not induce HI or virus-neutralizing antibodies against the H5, H7, or H9 influenza viruses. The challenge with these viruses significantly decreased the lung viral titers in BALB/c mice [115]. Several studies have demonstrated the protective effects of HA stem immunity through non-neutralizing, Fc-mediated effector functions, such as ADCC [116,117] and NP-specific cross-protective T-cell immune responses [118,119]. Since many Ad vectors have an inherent tropism to the respiratory tract, they are best suited for mucosal immunization [120]. The mucosal immunization stimulates robust IgA and CD8 T-cell responses and provides better protection than the intramuscular route [111,121,122,123].

Several nonhuman Ads are potential candidates for the new generation vaccine platform. One of the widely used platforms is the chimpanzee Ad (ChAd) vector. Some ChAd vector vaccines have already been licensed or evaluated in clinical trials for diseases such as SARS-CoV-2, Ebola virus, and hepatitis C [124,125,126]. Several researchers have improved the vaccine strategy by prime-boost with ChAd and modified vaccinia Ankara (MVA) vector vaccines. Three heterologous domains of M2e fused with NP were expressed in a ChAd vector [127]. The vaccine induced M2e-specific antibodies and NP-specific CD8+ T cell immune responses. Due to the availability of various types of Ads, the induction of robust humoral and cell-mediated immunity, and the choice of mucosal or systemic route, Ad vector platforms have considerable promise for developing a universal influenza vaccine. However, prime-boost immunization of mice with two types of ChAd showed partial protection after challenge with homologous influenza viruses [127]. The ChAd and MVA vector vaccines containing a combination of chimeric HA, NP, and M1 of the A(H3N2) virus were used in a prime (ChAd)-boost (MVA) approach [128]. The approach elicited higher levels of antibodies and IFN-γ-expressing CD8+ T cells against the expressed antigens [129], conferring complete protection following challenge with heterologous HA group 2 influenza viruses in BALB/c mice [128]. Similarly, the MVA vector system provides an excellent opportunity for designing broadly protective influenza vaccines.

MVA vector expressing NP alone or co-expressing other proteins (HA stems and M2e) induced significantly higher antibody and T cell responses against homosubtypic and heterosubtypic influenza viruses, leading to protection against A(H5N1), A(H7N1), and A(H9N2) influenza viruses with decreased weight loss and symptom scores in immunized BALB/c mice [130]. Another study expressed a mosaic HA in the MVA vector (MVA-H5M), and the BALB/c mice immunized were protected from challenge with a heterologous high-pathogenic A(H5N1) influenza virus [131]. However, the same vaccine showed reduced protection when immunized mice were challenged with a seasonal A(H1N1) influenza virus [131]. This vaccine failed to provide broader protection against all influenza strains.

## 7. Nucleic Acid-Based Vaccines

These are divided into two categories, DNA- and mRNA-based vaccines. Generally, a DNA vaccine is based on a plasmid encoding the gene of the antigen of interest under a eukaryotic promoter. Following immunization with a DNA vaccine, the protein of interest will be expressed and processed by antigen-presenting cells, leading to antigen-specific humoral and cellular immunity [132,133]. A critical factor that influences the efficacy of DNA vaccines is the delivery system, such as a gene gun, biodegradable skin patch, electroporation, or nanoparticles with polymers, lipids, or other molecules [133,134,135,136]. DNA vaccines are relatively simple to manufacture, stable at room temperature, and may contain gene construct representing multiple variants [137,138,139]. The currently licensed DNA vaccines are for veterinary use [140].

The generation of consensus sequences from 2656 full-length H1 HA sequences following the COBRA strategy was used to optimize the integration of T- and B-cell epitopes in developing a consensus DNA vaccine (pCH1) [141]. The pCH1 vaccine elicited broad humoral and cell-mediated immune responses against several A(H1N1) reassortant viruses, except for the A(H1N1)pdm09. Immunization of BALB/c mice with a combined vaccine containing pCH1 and the plasmid containing the A(H1N1)pdm09 HA gene decreased the histopathology lesions and provided complete protection against several A(H1N1) viruses, including A(H1N1)pdm09 [141]. A similar strategy was adopted for A(H5N1) influenza viruses [142]. The vaccine elicited significant neutralizing antibodies against various A(H5N1) viruses from different clades and protected mice from lethal challenges with reassortant A(H5N1) viruses [142].

To fully cover a broad range of strains, a modified strategy called micro-consensus was developed, representing four micro-consensus sequences (Figure 3E) from a phylogenetic tree of H1 viruses, thereby generating a vaccine cocktail [143]. Three doses of the micro-consensus vaccine by electroporation resulted in protective HI titers (≥1:40) against several seasonal and pandemic A(H1N1)pdm09 influenza viruses in both guinea pigs and nonhuman primates. It also reduced the weight loss (3% on average) and conferred complete protection against a lethal dose of an A(H1N1)pdm09 virus in ferrets, while 75% of the control animals died after the challenge [143]. The same strategy was exploited to develop a cocktail DNA vaccine based on the four micro-consensus sequences from the H3 subtype [144]. Robust humoral responses against eight A(H3N2) strains and HA-specific cellular immune responses were elicited in immunized BALB/c mice. There was no mortality and less than 10% weight loss in vaccinated mice after challenges with distinct A(H3N2) viruses [144]. In light of the success of the consensus HA subtype-specific sequence strategy for DNA vaccines, the capacity of this system to deliver more influenza subtypes in a cocktail vaccine for a universal influenza vaccine would be worth investigating.

Recently, mRNA vaccines have become the focus of a new vaccine technology due to the broad adoption of this technology for SARS-CoV-2 vaccines [145]. Transient expression of mRNA presents a safer candidate for vaccine development [146,147]. The lipid nanoparticles (LNPs) serve as a delivery vehicle for mRNA-based vaccines [148,149]. The conserved domains of influenza viruses were also the target antigens for mRNA vaccines. The mRNAs representing the HA stem from an A(H1N1) seasonal strain, and NA, NP, and M2 from an A(H1N1)pdm09 pandemic strain, were encapsulated in LNPs [150]. A single intradermal vaccination of BALB/c mice led to robust humoral and cell-mediated immune responses against target antigens. The combination of the four components protected against several group 1 influenza viruses, including A(H1N1), A(H5N8), and recombinant cH6/1N5 (chimeric avian H6 head domain on A(H1N1)pdm09 stalk domain coupled with an avian NA5 in the A/Puerto Rico/8/34(H1N1) [PR8] backbone), and showed lower morbidity compared to the vaccine containing a single component [150]. Immunization of mice or ferrets with A(H1N1)pdm09 HA mRNA vaccine elicited HA stem-specific antibodies leading to protection against homologous and heterologous H1 and H5 viruses [151]. A self-amplifying mRNA (SAM) vaccine encoding NP and/or M1 sequences in LNPs induced strong T cell immunity in BALB/c mice [152]. Central memory (TCM) and effector memory (TEM) CD4 and CD8 T cells were expanded. There was lower mortality, morbidity, and lung pathology in immunized mice following challenges with homologous or heterosubtypic influenza viruses [152].

## 8. New Vaccine Strategies on Existing Platforms

### 8.1. Chimeric HA

Sequential chimeric HA (cHA) is a potential strategy for developing a universal influenza vaccine (Figure 3A). The chimeric HA is designed to have a similar stalk domain with irrelevant head domains by swapping the original globular head domain between Cys52 and Cys277 with the head domain from another HA [153]. The first vaccination of naive animals with cHA led to predominately head domain-specific immune responses with a lower level of the stalk domain-specific immune responses [154,155]. However, subsequent immunization with the cHA containing the same stalk but a different head domain elicited a higher immune response against the stalk domain due to a memory response [154,155]. The anti-stalk immunity can be enhanced by repeating this procedure, leading to a broad cross-protection [154,155,156]. The group 1 cHAs as a universal vaccine is currently in Phase I clinical trial [54]. The cHAs are designed to carry the head domain from H8 and H5 with the H1 stalk, and the inactivated split viral vaccine was produced via reverse genetics in the PR8 backbone. The volunteers vaccinated with the inactivated cHA vaccine containing the AS03 adjuvant elicited higher titers of stalk-specific antibodies with cross-reactivity against several heterologous group 1 HA, including H2, H9, and H18 [54]. Adoptive transfer of the immunized volunteers’ serum to mice provided broad protection, as indicated by the reduction in weight loss following the challenge [54]. A similar strategy was also utilized for group 2 and influenza B HAs [157]. The BALB/c mice immunized with a chimeric H3 stalk vaccine by priming with a DNA vaccine and boosting with a subunit vaccine showed reductions in morbidity and mortality following challenge with the A(H3N2) or A(H7N1) virus [157]. The sera from immunized mice showed broad cross-reactivity with group 2 HA, including H3, H7, and H10 [157]. Since influenza B does not have HA subtypes, the exotic influenza A HA head is the only option for the cHA strategy for influenza B viruses. However, the chimeric influenza B virus could not be rescued, possibly due to the incompatibility between the head and stalk regions from different genera [158]. Therefore, an alternative strategy called “mosaic HA” (Figure 3B) was introduced by replacing the major antigenic sites of the influenza B HA head with amino acid residues from H5, H8, H11, or H13 [159]. The chimeric influenza B viruses containing mosaic HA replicated well in the embryonic chicken eggs. The sequential vaccination strategy with chimeric influenza B viruses in mice showed sufficient protection against the influenza B virus [159].

### 8.2. Hyperglycosylation of HA

Antigenic sites on the HA head can be shielded by glycosylation (Figure 3C) [160,161,162]. The masking of immunodominant antigenic sites may redirect the antibodies to the other sites. Seven additional N-linked glycosylation sites in the HA head domain of PR8 were added, and the hyperglycosylated HA was expressed in mammalian cells for a subunit vaccine [49]. The mouse group that received three immunizations with hyperglycosylated HA elicited nine-fold higher anti-stalk antibodies than wild-type HA [49]. The vaccinated mice showed lower morbidity and no mortality after the challenge with 20 lethal doses of the recombinant chimeric H9/1 virus (H9 head with H1 stalk).

### 8.3. M2-Modified Live Attenuated Influenza Vaccine (LAIV)

A live single replication influenza virus, M2SR, was generated by deleting the M2 transmembrane domain and inserting stop codons in the remaining M2 ORF in the PR8 virus [163]. The vaccinated mice showed complete protection against homosubtypic [A(H1N1)] and heterosubtypic [A(H3N2)] challenges. A virus-specific solid T cell response and systemic and mucosal antibody responses were observed in the vaccinated mice [163]. Subsequent studies replaced the HA and NA sequences of M2SR with that of the A(H5N1) virus and conferred protection and immunogenicity against the A(H5N1) virus in ferrets [164]. Similarly, an attenuated M2-mutated virus, W7–791, has a single mutation on 791 of the M gene sequence, and the other seven segments from the A/WSN/1933(H1N1) virus replicated well and were highly attenuated [165]. The vaccinated mice showed lower weight loss following the challenge with A(H1N1), A(H3N2), and A(H5N1) influenza viruses [165]. There was reduced viral shedding in immunized ferrets following challenges with A(H1N1) and A(H3N1) influenza viruses [165]. A LAIV containing four copies of M2e in front of A(H3N2) HA in the backbone of PR8 was used as a vaccine in mice [166]. The vaccinated mice showed no mortality against heterosubtypic A(H1N1), A(H5N1), A(H7N9), and A(H9N2) viruses, and the protection was better than the LAIV without 4xM2e [166]. A similar remodified structure of HA- 4xM2e was also used in another study. In a passive immunization trial, the serum from immunized BALB/c mice provided superior protection against heterologous viruses. This result indicated that the increased M2e-specific immune response plays a role in cross-protection [167].

### 8.4. Epitope-Based Influenza Vaccine

Epitope-targeting strategies have been used to generate universal influenza subunit vaccines by exploiting proteins or peptides consisting of epitopes critical for eliciting broadly protective cellular and humoral immunity [168]. A recombinant protein vaccine, M-001, containing nine conserved B and T cell epitopes from the HA, NP, and M1 of influenza A and B viruses with complete Freund’s adjuvant stimulated both cellular and humoral immune responses in mice, leading to reduced mortality following the heterosubtypic A(H5N1) challenge [169,170]. The M-001 vaccine elicited influenza-specific, cell-mediated immunity and increased the proportion of protective CD4+ lymphocytes in humans [168,171]. Another peptide vaccine based on six consensus peptides representing internal proteins of influenza A and B viruses with incomplete Freund’s adjuvant resulted in higher levels of IFN-γ excretion from the splenocytes of vaccinated mice when cocultured with human cells infected with three non-related influenza virus strains, indicating that cross-reactive immunity can be generated by stimulating conserved epitopes [172]. The vaccinated mice showed a lower mortality rate when challenged with a lethal influenza A virus [172]. The Phase Ib clinical trial showed increased cellular immunity with the expression of IFN-γ in the immunized volunteers demonstrating immunogenicity in humans. The Phase IIb trial demonstrated reduced influenza disease severity in the group immunized with 500 μg FLU-v as well [173,174].

Instead of targeting the epitopes of multiple influenza proteins, some studies have focused on the conserved region of the HA stem. The long α-helix (LAH, amino acids 76–130) region of the HA2 of A(H3) influenza virus was synthesized and coupled to a carrier protein with complete or incomplete Freund’s adjuvant as a peptide vaccine [175]. The serum from vaccinated mice showed cross-reactivity with several heterosubtypic influenza viruses, resulting in a moderate decline in mortality following challenge with A(H3) and A(H5) viruses [175]. Similarly, another study analyzed the conserved peptide sequences of HA stem from the database and predicted the potential T and B cell epitopes for a peptide vaccine with complete or incomplete Freund’s adjuvant [176]. The serum from vaccinated mice showed neutralizing activities against A(H1N1) and A(H3N2) viruses [176]. Alternatively, the fusion of two tandem copies of consensus M2e sequence from human influenza A and two copies of M2e from A(H5N1) viruses and flagellin, which exhibited strong adjuvant properties, were used as a peptide vaccine [177]. The immunized mice showed decreased mortality and lung viral load when challenged with A(H1N1)pdm09, A(H3N2), and A(H5N1) viruses [177]. Intranasally vaccinated BALB/c mice with the improved 4M2e-HA2-flagellin vaccine showed significant mucosal and systemic immune responses [178]. Moreover, a higher survival rate was observed following the challenge compared to the original M2e vaccine design [178,179]. One of the critical limitations of peptide vaccines is poor immunogenicity [180]. Improved adjuvants, immunostimulators, or optimal delivery routes are required to stimulate the appropriate immunity of peptide vaccines [180,181]. The short peptides usually present only linear epitopes, primarily targeting T cells, while many B cell epitopes are conformational [182]. It is necessary to consider the peptide secondary structure via computational prediction to improve the induction of specific humoral responses.

## 9. Conclusions

Influenza viruses hugely impact public health, and vaccination is considered as the best strategy to control the disease. Currently, the licensed seasonal influenza vaccines are strain-specific. Due to the frequent antigen drift and shift in the influenza viruses, more effort is needed in disease surveillance to decide the appropriate strains for the seasonal influenza vaccines. Once a mismatched strain emerges, the vaccine provides only partial or no protection. The narrow range of protection implies that current influenza vaccines lack a prompt response to the emerging outbreaks, and the occurrence of numerous human infections by zoonotic influenza viruses reflects the need for a universal influenza vaccine.

Most universal influenza vaccine candidates target the conserved antigenic domains of the influenza virus such as HA stem, M2e, NP, and relatively conserved NA to provide broader protection. Several strategies are utilized to evade the immunodominance of the HA head domain and elicit robust immunity against the conserved targets. Sequential chimeric HA and mosaic HA lead to cumulative immunity toward the HA stem. Hyperglycosylation of HA decreases the immune response to the head domain and enhances the response to the stem domain. In addition, several innovative platforms have been developed to make better immune responses than conventional influenza vaccines. VLPs can present the antigens in their natural conformation, resulting in a better immune response. Nanoparticles are valuable platforms to express the antigens in high density and provide adjuvant-like functions. Viral vector vaccines stimulate robust humoral and cellular immunity and can be delivered by the systemic or mucosal route. The nucleic acid platform, which showed a tremendous contribution to the COVID-19 pandemic, is an innovative tool to manipulate the antigen easily, and it has the potential to respond quickly to an emerging outbreak. Several studies have shown broad protection against multiple influenza viruses, and some are under preclinical or clinical trials. However, more studies are needed to develop effective universal influenza vaccines.

## 10. Future Direction

The frequently emerging zoonotic influenza viruses and the recent outbreak of the SARS-CoV-2 pandemic have further emphasized the urgent need for a universal influenza vaccine. Developing the strain-specific vaccine early enough to control a pandemic would be difficult. Several concerns hinder the development of the universal influenza vaccine. First, the seasonal influenza vaccines are manufactured in a well-established system, and several companies can produce them on a large scale. Since the current vaccine can still provide adequate protection with low mortality, it would be a huge challenge to convince companies to contribute enormous funding to a brand-new-designed vaccine. As in the case of a pandemic, universal influenza vaccine development will have to be a government-funded operation. Second, most of the universal influenza vaccine studies were currently restricted to heterosubtypic protection. Due to the differences in the genome between influenza A and B, the strategies focusing on a single conserved domain may not provide universal protection from all influenza types. The vaccine should include the essential immunogens from influenza A and B viruses. The platform should be able to express multiple immunogens and potentially adopt them in the design.

Additionally, several studies have demonstrated the stimulation of cellular immunity and non-neutralizing antibodies’ role in the protection. The viral vector-based and mRNA platforms induce robust cellular and humoral immunity and are promising expression systems for future vaccines. Moreover, evaluating vaccine efficacy based only on neutralizing antibody titers is insufficient to assess the breadth of universal influenza vaccines. Protection correlates assessing cellular immunity and non-neutralizing antibody levels need to be included in the evaluation. Leveraging the expedited progress of the vaccine development, more investigations into the influenza vaccines should be targeted toward a universal influenza vaccine.

## Figures and Tables

**Table 1:** Universal influenza vaccines in clinical trials.

Platform	Vaccine Type	Target Antigen	Stage	Trial ID
LAIV/Inactivated virus	Single-replication virus	Whole virus (M2-deleted)	Phase I	NCT04960397 NCT02822105 NCT03999554
	Inactivated split virus	HA stem (chimeric)	Phase I	NCT03275389
	Inactivated whole virus	Whole virus	Phase I	NCT05027932
	LAIV + Inactivated split virus	HA stem (chimeric)	Phase I	NCT03300050
Subunit vaccine	Recombinant protein	M1, NP, HA	Phase I, II, III	NCT01419925 NCT00877448 NCT02293317 NCT03450915 NCT01146119 NCT02691130
	Recombinant protein	M2e	Phase I, II	NCT00921947 NCT00921973 NCT00921206 NCT00603811
	Synthetic peptides	NP, M, PB1, PB2	Phase I	NCT01265914
	Synthetic peptides	M1, M2, NP	Phase II	NCT03180801 NCT02962908 NCT01226758 NCT01181336
VLP/Nanoparticle	Ferritin-based nanoparticles	HA stem	Phase I	NCT05155319
	Ferritin-based nanoparticles	HA stem	Phase I	NCT04579250
	Computational design nanoparticles	HA	Phase I	NCT04896086
	Oligomerization domain-based nanoparticles	NP	Phase II	NCT04192500
	Hepatitis B VLP	M2e	Phase I	NCT00819013
	Hepatitis B VLP	M2e	Phase I	NCT03789539
Viral vector	MVA	NP, M1	Phase II	NCT03880474 NCT03883113 NCT00993083
	ChAd + MVA	NP, M1	Phase I	NCT01818362 NCT01623518
Nucleic acid	DNA	HA, NA, M2e, NP	Phase I	NCT01184976

LAIV: live attenuated influenza virus; VLP: virus-like particle; MVA: Modified vaccinia Ankara; ChAd: chimpanzee adenovirus.

## Data Availability

Not applicable.
